# Effectiveness of BNT162b2 (Pfizer-BioNTech) mRNA Vaccination Against Multisystem Inflammatory Syndrome in Children Among Persons Aged 12–18 Years — United States, July–December 2021

**DOI:** 10.15585/mmwr.mm7102e1

**Published:** 2022-01-14

**Authors:** Laura D. Zambrano, Margaret M. Newhams, Samantha M. Olson, Natasha B. Halasa, Ashley M. Price, Julie A. Boom, Leila C. Sahni, Satoshi Kamidani, Keiko M. Tarquinio, Aline B. Maddux, Sabrina M. Heidemann, Samina S. Bhumbra, Katherine E. Bline, Ryan A. Nofziger, Charlotte V. Hobbs, Tamara T. Bradford, Natalie Z. Cvijanovich, Katherine Irby, Elizabeth H. Mack, Melissa L. Cullimore, Pia S. Pannaraj, Michele Kong, Tracie C. Walker, Shira J. Gertz, Kelly N. Michelson, Melissa A. Cameron, Kathleen Chiotos, Mia Maamari, Jennifer E. Schuster, Amber O. Orzel, Manish M. Patel, Angela P. Campbell, Adrienne G. Randolph, Overcoming COVID-19 Investigators, Overcoming COVID-19 Investigators, Meghan Murdock, Mary Glas Gaspers, Katri V. Typpo, Connor P. Kelley, Ronald C. Sanders, Masson Yates, Chelsea Smith, Katheryn Crane, Geraldina Lionetti, Juliana Murcia-Montoya, Matt S. Zinter, Denise Villarreal-Chico, Adam L. Skura, Harvey Peralta, Justin M. Lockwood, Emily Port, Imogene A. Carson, Brandon M. Chatani, Laila Hussaini, Nadine Baida, Bria M. Coates, Courtney M. Rowan, Mary Stumpf, Marla S. Johnston, Benjamin J. Boutselis, Suden Kucukak, Sabrina R. Chen, Edie Weller, Laura Berbert, Jie He, Heidi R. Flori, Janet R. Hume, Ellen R. Bruno, Lexie A. Goertzen, Emily R. Levy, Supriya Behl, Noelle M. Drapeau, Lora Martin, Lacy Malloch, Cameron Sanders, Kayla Patterson, Anita Dhanrajani, Shannon M. Hill, Abigail Kietzman, Valerie H. Rinehart, Lauren A. Hoody, Stephanie P. Schwartz, Angelo G. Navas, Paris C. Bennett, Nicole A. Twinem, Merry L. Tomcany, Mary Allen Staat, Chelsea C. Rohlfs, Amber Wolfe, Rebecca L. Douglas, Kathlyn Phengchomphet, Megan M. Bickford, Lauren E. Wakefield, Laura Smallcomb, Laura S. Stewart, Meena Golchha, Jennifer N. Oates, Cindy Bowens

**Affiliations:** ^1^CDC COVID-19 Response Team; ^2^Department of Anesthesiology, Critical Care, and Pain Medicine, Boston Children’s Hospital, Boston, Massachusetts; ^3^Division of Pediatric Infectious Diseases, Department of Pediatrics, Vanderbilt University Medical Center, Nashville, Tennessee; ^4^Department of Pediatrics, Baylor College of Medicine, Immunization Project, Texas Children’s Hospital, Houston, Texas; ^5^The Center for Childhood Infections and Vaccines of Children’s Healthcare of Atlanta and the Department of Pediatrics, Emory University School of Medicine, Atlanta, Georgia; ^6^Division of Critical Care Medicine, Department of Pediatrics, Emory University School of Medicine, Children's Healthcare of Atlanta, Atlanta, Georgia; ^7^Department of Pediatrics, Section of Critical Care Medicine, University of Colorado School of Medicine and Children's Hospital Colorado, Aurora, Colorado; ^8^Department of Pediatrics, Children’s Hospital of Michigan, Central Michigan University, Detroit, Michigan; ^9^The Ryan White Center for Pediatric Infectious Disease and Global Health, Department of Pediatrics, Indiana University School of Medicine, Indianapolis, Indiana; ^10^Division of Pediatric Critical Care Medicine, Nationwide Children’s Hospital Columbus, Ohio; ^11^Division of Critical Care Medicine, Department of Pediatrics, Akron Children’s Hospital, Akron, Ohio; ^12^Department of Pediatrics, Department of Microbiology, Division of Infectious Diseases, University of Mississippi Medical Center, Jackson, Mississippi; ^13^Department of Pediatrics, Division of Cardiology, Louisiana State University Health Sciences Center and Children’s Hospital of New Orleans, New Orleans, Louisiana; ^14^Division of Critical Care Medicine, UCSF Benioff Children's Hospital Oakland, Oakland, California; ^15^Section of Pediatric Critical Care, Department of Pediatrics, Arkansas Children's Hospital, Little Rock, Arkansas; ^16^Division of Pediatric Critical Care Medicine, Medical University of South Carolina, Charleston, South Carolina; ^17^Division of Pediatric Critical Care, Department of Pediatrics, Children’s Hospital and Medical Center, Omaha, Nebraska; ^18^Division of Infectious Diseases, Children’s Hospital Los Angeles and Departments of Pediatrics and Molecular Microbiology and Immunology, University of Southern California, Los Angeles, California; ^19^Division of Pediatric Critical Care Medicine, Department of Pediatrics, University of Alabama at Birmingham, Birmingham, Alabama; ^20^Department of Pediatrics, University of North Carolina at Chapel Hill Children's Hospital, Chapel Hill, North Carolina; ^21^Division of Pediatric Critical Care, Department of Pediatrics, Saint Barnabas Medical Center, Livingston, New Jersey; ^22^Division of Critical Care Medicine, Department of Pediatrics, Northwestern University Feinberg School of Medicine, Ann & Robert H. Lurie Children's Hospital of Chicago, Chicago, Illinois; ^23^Division of Pediatric Hospital Medicine, UC San Diego-Rady Children’s Hospital, San Diego, California; ^24^Division of Critical Care Medicine, Department of Anesthesiology and Critical Care, Children’s Hospital of Philadelphia, Philadelphia, Pennsylvania; ^25^Department of Pediatrics, Division of Critical Care Medicine, University of Texas Southwestern, Children's Medical Center Dallas, Dallas, Texas; ^26^Division of Pediatric Infectious Diseases, Department of Pediatrics, Children’s Mercy Kansas City, Kansas City, Missouri; ^27^Departments of Anesthesia and Pediatrics, Harvard Medical School, Boston, Massachusetts.; Children’s of Alabama, Birmingham, Alabama; University of Arizona, Tucson, Arizona; University of Arizona, Tucson, Arizona; University of Arizona, Tucson, Arizona; Arkansas Children’s Hospital, Little Rock, Arkansas; Arkansas Children’s Hospital, Little Rock, Arkansas; Arkansas Children’s Hospital, Little Rock, Arkansas; Rady Children’s Hospital, San Diego, California; University of California, San Francisco Benioff Children’s Hospital Oakland, Oakland, California; University of California, San Francisco Benioff Children’s Hospital Oakland, Oakland, California; University of California; San Francisco Benioff Children’s Hospital, San Francisco, California; University of California; San Francisco Benioff Children’s Hospital, San Francisco, California; Children’s Hospital Los Angeles, Los Angeles, California; Children’s Hospital Los Angeles, Los Angeles, California; Children’s Hospital Colorado, Aurora, Colorado; Children’s Hospital Colorado, Aurora, Colorado; Children’s Hospital Colorado, Aurora, Colorado; Holtz Children’s Hospital, Miami, Florida; Emory University School of Medicine; Children’s Healthcare of Atlanta, Atlanta, Georgia; Emory University School of Medicine; Children’s Healthcare of Atlanta, Atlanta, Georgia; Ann & Robert H. Lurie Children’s Hospital of Chicago, Chicago, Illinois; Riley Hospital for Children, Indianapolis, Indiana; Riley Hospital for Children, Indianapolis, Indiana; Children’s Hospital of New Orleans, New Orleans, Louisiana; Boston Children’s Hospital, Boston, Massachusetts; Boston Children’s Hospital, Boston, Massachusetts; Boston Children’s Hospital, Boston, Massachusetts; Boston Children’s Hospital, Boston, Massachusetts; Boston Children’s Hospital, Boston, Massachusetts; Boston Children’s Hospital, Boston, Massachusetts; University of Michigan CS Mott Children’s Hospital, Ann Arbor, Michigan; University of Minnesota Masonic Children’s Hospital, Minneapolis, Minnesota; University of Minnesota Masonic Children’s Hospital, Minneapolis, Minnesota; University of Minnesota Masonic Children’s Hospital, Minneapolis, Minnesota; Mayo Clinic, Rochester, Minnesota; Mayo Clinic, Rochester, Minnesota; Mayo Clinic, Rochester, Minnesota; Children’s Hospital of Mississippi, Jackson, Mississippi; Children’s Hospital of Mississippi, Jackson, Mississippi; Children’s Hospital of Mississippi, Jackson, Mississippi; Children’s Hospital of Mississippi, Jackson, Mississippi; Children’s Hospital of Mississippi, Jackson, Mississippi; Children’s Mercy Hospital, Kansas City, Missouri; Children’s Mercy Hospital, Kansas City, Missouri; Children’s Hospital & Medical Center, Omaha, Nebraska; Children’s Hospital & Medical Center, Omaha, Nebraska; University of North Carolina at Chapel Hill, Chapel Hill, North Carolina; University of North Carolina at Chapel Hill, Chapel Hill, North Carolina; University of North Carolina at Chapel Hill, Chapel Hill, North Carolina; Akron Children’s Hospital, Akron, Ohio; Akron Children’s Hospital, Akron, Ohio; Cincinnati Children’s Hospital, Cincinnati, Ohio; Cincinnati Children’s Hospital, Cincinnati, Ohio; Nationwide Children’s Hospital, Columbus, Ohio; Children’s Hospital of Philadelphia, Philadelphia, Pennsylvania; Children’s Hospital of Philadelphia, Philadelphia, Pennsylvania; Medical University of South Carolina Children’s Health, Charleston, South Carolina; Medical University of South Carolina Children’s Health, Charleston, South Carolina; Medical University of South Carolina Children’s Health, Charleston, South Carolina; Monroe Carell Jr. Children’s Hospital at Vanderbilt, Nashville, Tennessee; Monroe Carell Jr. Children’s Hospital at Vanderbilt, Nashville, Tennessee; Texas Children’s Hospital, Houston, Texas; University of Texas Southwestern, Children’s Medical Center Dallas, Dallas, Texas

Multisystem inflammatory syndrome in children (MIS-C) is a severe postinfectious hyperinflammatory condition, which generally occurs 2–6 weeks after a typically mild or asymptomatic infection with SARS-CoV-2, the virus that causes COVID-19 (*1*–*3*). In the United States, the BNT162b2 (Pfizer-BioNTech) COVID-19 vaccine is currently authorized for use in children and adolescents aged 5–15 years under an Emergency Use Authorization and is fully licensed by the Food and Drug Administration for persons aged ≥16 years (*4*). Prelicensure randomized trials in persons aged ≥5 years documented high vaccine efficacy and immunogenicity (*5*),^§^ and real-world studies in persons aged 12–18 years demonstrated high vaccine effectiveness (VE) against severe COVID-19 (*6*). Recent evidence suggests that COVID-19 vaccination is associated with lower MIS-C incidence among adolescents (*7*); however, VE of the 2-dose Pfizer-BioNTech regimen against MIS-C has not been evaluated. The effectiveness of 2 doses of Pfizer-BioNTech vaccine received ≥28 days before hospital admission in preventing MIS-C was assessed using a test-negative case-control design^¶^ among hospitalized patients aged 12–18 years at 24 pediatric hospitals in 20 states** during July 1–December 9, 2021, the period when most MIS-C patients could be temporally linked to SARS-CoV-2 B.1.617.2 (Delta) variant predominance. Patients with MIS-C (case-patients) and two groups of hospitalized controls matched to case-patients were evaluated: test-negative controls had at least one COVID-19–like symptom and negative SARS-CoV-2 reverse transcription–polymerase chain reaction (RT-PCR) or antigen-based assay results, and syndrome-negative controls were hospitalized patients without COVID-19–like illness. Among 102 MIS-C case-patients and 181 hospitalized controls, estimated effectiveness of 2 doses of Pfizer-BioNTech vaccine against MIS-C was 91% (95% CI = 78%–97%). All 38 MIS-C patients requiring life support were unvaccinated. Receipt of 2 doses of the Pfizer-BioNTech vaccine is associated with a high level of protection against MIS-C in persons aged 12–18 years, highlighting the importance of vaccination among all eligible children.

This study used a test-negative case-control design, commonly used for postauthorization VE evaluations (*6*,*8*). Patients were hospitalized at 24 participating sites in the Overcoming COVID-19 Network, a collaboration between CDC and approximately 70 pediatric hospitals nationwide to assess COVID-19 complications in children and young adults.^††^ Given that children aged 5–11 years were not recommended to receive the Pfizer-BioNTech vaccine until November 2, 2021,^§§^ this analysis focused on persons aged 12–18 years.^¶¶^ VE was assessed by comparing the odds of antecedent vaccination between MIS-C patients and hospitalized controls without evidence of SARS-CoV-2 infection during July 1–December 9, 2021. Case-patients met CDC criteria for MIS-C,*** which included a clinically severe illness requiring hospitalization, temperature ≥100.4°F (38°C) for ≥24 hours or subjective fever, evidence of inflammation (demonstrated by elevated levels of inflammatory markers), involvement of two or more organ systems, no alternative plausible diagnosis, and current or recent SARS-CoV-2 infection, indicated by a positive result from an RT-PCR test, serologic test, or antigen test. Two hospitalized control groups included 1) patients with one or more symptoms consistent with COVID-19, but with a negative result from a SARS-CoV-2 RT-PCR or antigen test (test-negative) and 2) patients without symptoms compatible with COVID-19 who might or might not have received SARS-CoV-2 testing (syndrome-negative).^†††^ Eligible controls were matched to case-patients by site, age group (12–15 years and 16–18 years), and case-patient hospitalization date (within plus or minus approximately 3 weeks).

Vaccination status was verified through searches of state immunization information systems, electronic medical records, or other sources, including documentation from pediatricians or patient immunization cards. For this analysis, persons were categorized as unvaccinated or fully vaccinated on or before the case-patient hospitalization date. Patients were considered unvaccinated if they had received no doses of the Pfizer-BioNTech vaccine; full vaccination in terms of expected protection against MIS-C was defined as receipt of 2 doses of Pfizer-BioNTech COVID-19 vaccine, with receipt of the second dose ≥28 days before hospital admission. The 28-day window was selected because a person is considered fully vaccinated against COVID-19 ≥14 days after receipt of the second dose, and MIS-C generally occurs approximately 2–6 weeks after SARS-CoV-2 infection, with most cases occurring by the fourth week (*1*–*3*). Patients were excluded based on the following conditions: 1) receipt of only 1 vaccine dose; 2) receipt of the second dose within 28 days of hospital admission; 3) age 12–15 years and admission before July 1, 2021 (given that vaccination was not expanded to this age group until May 12, 2021); and 4) receipt of any COVID-19 vaccine other than Pfizer-BioNTech.

Demographic characteristics, clinical information related to the current illness, and SARS-CoV-2 testing history were obtained through parent or guardian interview conducted by trained study personnel or review of electronic medical records.^§§§^ Descriptive statistics were used to compare characteristics of case-patients and hospitalized controls, and Fisher’s exact or Wilcoxon rank-sum tests were used for categorical and continuous variables, respectively. VE against MIS-C was calculated by comparing the odds of full COVID-19 vaccination among MIS-C case-patients and controls using the equation VE = 100 X (1 − adjusted odds ratio). Adjusted odds ratios were calculated using multivariable logistic regression models with Firth penalization to reduce bias contributed by sparse data. Models were adjusted for U.S. Census region, age, sex, and race/ethnicity (*8*). To account for potential residual confounding by calendar time related to increasing vaccination coverage, the case-patient hospitalization date was used as a reference point for comparing antecedent vaccination in case-patients and controls. Other factors (underlying health conditions and social vulnerability index) were assessed, but not included in the final model if they did not alter the odds ratio estimate by >5%. Sensitivity analyses were conducted to evaluate VE against MIS-C among patients with serologic evidence of previous infection (because non–MIS-C acute COVID-19 patients might have a positive RT-PCR assay in the absence of serology) and to evaluate whether VE differed by control group. Statistical analyses were conducted using SAS (version 9.4; SAS Institute); statistical significance was defined as p<0.05. This activity was reviewed by CDC and other participating institutions and was conducted consistent with applicable federal law and CDC policy.^¶¶¶^

During July 1–December 9, 2021, among 117 MIS-C case-patients aged 12–18 years, 15 were excluded from the analysis, including six patients who received only 1 dose by the date of hospitalization, four who received their second vaccine dose within 28 days of hospital admission, and five patients aged 12–15 years who were hospitalized before July 1, 2021. The 283 patients in the primary analysis included 102 MIS-C case-patients and 181 controls (90 [50%] test-negative and 91 [50%] syndrome-negative) (Table 1). The median age among all case-patients and controls was 14.5 years, and 58% had at least one underlying condition (including obesity). COVID-19 vaccination coverage was approximately 5% among case-patients and 36% among controls.

**TABLE 1:** Characteristics of multisystem inflammatory syndrome in children case-patients and controls aged 12–18 years — 24 pediatric hospitals, 20 U.S. states,* July 1–December 9, 2021

Characteristic	No. (%)			
	Total	MIS-C case-patients	Controls	p-value^†^
	(N = 283)	(n = 102)	(n = 181)	
**Median age, yrs (IQR)**	14.5 (13.4–15.9)	14.2 (13.0–15.9)	14.7 (13.6–15.9)	0.06
**Age group, yrs**				
12–15	221 (78.1)	81 (79.4)	140 (77.3)	0.77
16–18	62 (21.9)	21 (20.6)	41 (22.7)	
**Sex**				
Female	132 (46.6)	30 (29.4)	102 (56.4)	<0.01
**Race/Ethnicity**				
White, non-Hispanic	105 (37.1)	32 (31.4)	73 (40.3)	0.39
Black, non-Hispanic	99 (35.0)	42 (41.2)	57 (31.5)	
Asian, non-Hispanic	8 (2.8)	1 (1.0)	7 (3.9)	
Hispanic, any race	51 (18.0)	19 (18.6)	32 (17.7)	
Multiple/Other, non-Hispanic	10 (3.5)	4 (3.9)	6 (3.3)	
Unknown	10 (3.5)	4 (3.9)	6 (3.3)	
**SVI,^§^ median (IQR)**	0.60 (0.30–0.80)	0.64 (0.43–0.78)	0.56 (0.27–0.81)	0.09
U.S. Census region*				
Northeast	8 (2.8)	3 (2.9)	5 (2.8)	0.98
Midwest	75 (26.5)	28 (27.5)	47 (26.0)	
South	159 (56.2)	56 (54.9)	103 (56.9)	
West	41 (14.5)	15 (14.7)	26 (14.4)	
**Month of admission**				
June	1 (0.4)	0 (—)	1 (0.6)	0.35
July	9 (3.2)	5 (4.9)	4 (2.2)	
August	49 (17.3)	16 (15.7)	33 (18.2)	
September	82 (29.0)	35 (34.3)	47 (26.0)	
October	85 (30.0)	30 (29.4)	55 (30.4)	
November	48 (17.0)	15 (14.7)	33 (18.2)	
December	9 (3.2)	1 (1.0)	8 (4.4)	
**Underlying health condition^¶^**				
At least one underlying condition (including obesity)	164 (58.0)	40 (39.2)	124 (68.5)	<0.01
Asthma	49 (17.3)	15 (14.7)	34 (18.8)	0.42
Cardiovascular system disorder	23 (8.1)	3 (2.9)	20 (11.0)	0.02
Neurologic/Neuromuscular disorder	45 (15.9)	7 (6.9)	38 (21.0)	<0.01
Active or previous oncologic disorder	9 (3.2)	1 (1.0)	8 (4.4)	0.16
Nononcologic immunosuppressive disorder	13 (4.6)	2 (2.0)	11 (6.1)	0.14
Endocrine disorder	16 (5.7)	4 (3.9)	12 (6.6)	0.43
Diabetes	9 (3.2)	2 (2.0)	7 (3.9)	0.50
Other chronic conditions**	97 (34.3)	21 (20.6)	76 (42.0)	<0.01
**Laboratory test results^††^**				
RT-PCR or antigen-positive, antibody not performed	11 (3.9)	11 (10.8)	0 (—)	<0.01
RT-PCR or antigen-positive, antibody-positive	12 (4.2)	12 (11.8)	0 (—)	
Antibody positive only	76 (26.9)	76 (74.5)	0 (—)	
Pre-admission results available only	3 (1.1)	3 (2.9)	0 (—)	
**Fully vaccinated^§§^**	70 (24.7)	5 (4.9)	65 (35.9)	<0.01
Median interval from receipt of second vaccine dose to reference hospitalization date, days (IQR)^¶¶^	84 (51–122)	63 (48–89)	88 (52–122)	0.37

**Abbreviations:** MIS-C = multisystem inflammatory syndrome in children; RT-PCR = reverse transcription-polymerase chain reaction; SVI = social vulnerability index.

* Patients included vaccinated and unvaccinated persons aged 12–18 years enrolled from 24 pediatric hospitals in 20 states. *Northeast:* Boston Children’s Hospital (Massachusetts), Children's Hospital of Philadelphia (Pennsylvania), and Saint Barnabas Medical Center (New Jersey); *Midwest:* Akron Children’s Hospital (Ohio), Children’s Hospital and Medical Center: Nebraska (Nebraska), Children's Hospital of Michigan (Michigan), Children’s Mercy Kansas City (Missouri), Cincinnati Children’s Hospital Medical Center (Ohio), Lurie Children's Hospital of Chicago (Illinois), Mayo Clinic (Minnesota), Nationwide Children's Hospital (Ohio), and Riley Children's Hospital (Indiana); *South:* Arkansas Children’s Hospital (Arkansas), Children’s of Alabama (Alabama), Children's Healthcare of Atlanta (Georgia), Children’s Hospital of New Orleans (Louisiana), Medical University of South Carolina Children’s Health (South Carolina), Monroe Carell Jr. Children’s Hospital at Vanderbilt (Tennessee), Texas Children’s Hospital (Texas), University of Mississippi Medical Center (Mississippi), University of North Carolina at Chapel Hill Children’s Hospital (North Carolina), and University of Texas Southwestern Medical Center (Texas); *West:* Children’s Hospital Colorado (Colorado), Children’s Hospital Los Angeles (California), University of California San Diego-Rady Children’s Hospital (California), and University of California San Francisco Benioff Children’s Hospital Oakland (California).

^†^ Testing for statistical significance was conducted using Fisher's exact test to compare categorical variables or Wilcoxon rank-sum test for medians to compare continuous data. Statistical significance was defined as p<0.05.

^§^ CDC/ATSDR SVI documentation is available at https://www.atsdr.cdc.gov/placeandhealth/svi/index.html. Median SVI for case-patients and controls are based on U.S. 2018 SVI data.

^¶^ Underlying conditions with a missing response (yes/no) were assumed not to be present.

** Other chronic conditions included rheumatologic/autoimmune disorder, hematologic disorder, renal or urologic dysfunction, gastrointestinal/hepatic disorder, metabolic or confirmed or suspected genetic disorder (including obesity), or atopic or allergic condition.

^††^ With the exception of the “pre-admission results available only” category, all other test results were obtained after hospital admission.

^§§^ COVID-19 vaccination status included the following two categories: 1) unvaccinated, defined as no receipt of any SARS-CoV-2 vaccine before hospitalization for current illness and 2) fully vaccinated, defined as receipt of both doses of a 2-dose Pfizer-BioNTech vaccination ≥28 days before illness onset.

^¶¶^ Dates are based on those with documented vaccination, not plausible self-report. For controls without COVID-19–like illness, a reference date was set to the admission date of their matched case-patient to account for residual confounding by hospital admission date relative to expanding vaccination coverage.

Among the 70 children in this analysis who were fully vaccinated (with 2 doses), one syndrome-negative control patient had received a third dose. Among 102 MIS-C case-patients, five (5%) were fully vaccinated with 2 doses ≥28 days before hospitalization, and 97 (95%) were unvaccinated (Table 2). Overall, 91 (89%) case-patients had cardiovascular involvement, 84 (82%) had gastrointestinal involvement, and 68 (67%) had hematologic involvement. Sixty-two (61%) were admitted to an intensive care unit, and 38 (37%) received life support during hospitalization, including invasive mechanical ventilation, vasoactive infusions, or extracorporeal membrane oxygenation (ECMO). All 38 MIS-C patients requiring life support were unvaccinated; among these, nine patients required invasive mechanical ventilation, 35 received vasoactive infusions and one required ECMO. No deaths among these patients were reported. Hospital length of stay was similar among vaccinated and unvaccinated MIS-C patients (median = 5 days).

**TABLE 2:** Clinical outcomes and severity among multisystem inflammatory syndrome in children case-patients aged 12–18 years, by vaccination status* — 24 pediatric hospitals, 20 U.S. states,^†^ July–December 2021

Characteristic	No. (%)		
	Total	Unvaccinated	Fully vaccinated ≥28 days before hospitalization
	(N = 102)	(n = 97)	(n = 5)
**Organ system involvement^§^**			
Cardiovascular	91 (89.2)	86 (88.7)	5 (100.0)
Respiratory	29 (28.4)	28 (28.9)	1 (20.0)
Hematologic	68 (66.7)	66 (68.0)	2 (40.0)
Gastrointestinal	84 (82.4)	79 (81.4)	5 (100.0)
Neurologic	9 (8.8)	8 (8.2)	1 (20.0)
Dermatologic	36 (35.3)	34 (35.1)	2 (40.0)
Renal/Urologic	35 (34.3)	33 (34.0)	2 (40.0)
**Intensive care unit admission**	62 (60.8)	61 (62.9)	1 (20.0)
**Critically ill patients on life support**	38 (37.3)	38 (39.2)	0 (—)
Invasive mechanical ventilation	9 (8.8)	9 (9.3)	0 (—)
Vasoactive infusions	35 (34.3)	35 (36.1)	0 (—)
Extracorporeal membrane oxygenation	1 (1.0)	1 (1.0)	0 (—)
**Patients with discharge data**	101 (99.0)	96 (99.0)	5 (100.0)
Hospital length of stay, median (IQR)	5 (4–8)	5 (4–8)	5 (2–6)

**Abbreviation:** BNP = brain natriuretic peptide.

* COVID-19 vaccination status included the following two categories: 1) unvaccinated, defined as no receipt of any SARS-CoV-2 vaccine before hospitalization for current illness and 2) fully vaccinated, defined as receipt of both doses of a 2-dose Pfizer-BioNTech vaccination ≥28 days before illness onset.

^†^ Patients included vaccinated and unvaccinated persons aged 12–18 years enrolled from 24 pediatric hospitals in 20 states. *Northeast:* Boston Children’s Hospital (Massachusetts), Children's Hospital of Philadelphia (Pennsylvania), and Saint Barnabas Medical Center (New Jersey); *Midwest:* Akron Children’s Hospital (Ohio), Children’s Hospital and Medical Center: Nebraska (Nebraska), Children's Hospital of Michigan (Michigan), Children’s Mercy Kansas City (Missouri), Cincinnati Children’s Hospital Medical Center (Ohio), Lurie Children's Hospital of Chicago (Illinois), Mayo Clinic (Minnesota), Nationwide Children's Hospital (Ohio), and Riley Children's Hospital (Indiana); *South:* Arkansas Children’s Hospital (Arkansas), Children’s of Alabama (Alabama), Children's Healthcare of Atlanta (Georgia), Children’s Hospital of New Orleans (Louisiana), Medical University of South Carolina Children’s Health (South Carolina), Monroe Carell Jr. Children’s Hospital at Vanderbilt (Tennessee), Texas Children’s Hospital (Texas), University of Mississippi Medical Center (Mississippi), University of North Carolina at Chapel Hill Children’s Hospital (North Carolina), and University of Texas Southwestern Medical Center (Texas); *West:* Children’s Hospital Colorado (Colorado), Children’s Hospital Los Angeles (California), University of California San Diego-Rady Children’s Hospital (California), and University of California San Francisco Benioff Children’s Hospital Oakland (California).

^§^ Organ system involvement was defined with the following criteria: 1) Cardiovascular (e.g., shock, elevated troponin, BNP, N-terminal-pro hormone BNP, abnormal echocardiogram, or arrhythmia); 2) Respiratory (e.g., pneumonia, acute respiratory distress syndrome, and pulmonary embolism); 3) Renal (e.g., acute kidney injury or renal failure); 4) Gastrointestinal (e.g., abdominal pain, vomiting, diarrhea, elevated bilirubin, or elevated liver enzymes); 5) Neurologic (e.g., cerebrovascular accident, aseptic meningitis, or encephalopathy); 6) Hematologic (e.g., elevated D-dimers, thrombophilia, or thrombocytopenia); 7) Dermatologic (e.g., rash, erythema, or peeling).

VE against MIS-C was 91% (95% CI = 78%–97%) (Table 3).**** In a sensitivity analysis excluding patients with positive RT-PCR or antigen-based SARS-CoV-2 test results and no positive serologic test, VE was 90% (95% CI = 75%–96%). VE against MIS-C was similar, irrespective of control group (test-negative controls: 92%, 95% CI = 77%–97%; syndrome-negative controls: 89%, 95% CI = 70%–96%); therefore, the pooled VE estimate using both control populations was deemed acceptable.

**TABLE 3:** Effectiveness* of 2 doses of Pfizer-BioNTech vaccine against multisystem inflammatory syndrome in children among hospitalized patients aged 12–18 years — 24 pediatric hospitals, 20 U.S. states,^†^ July–December 2021

Control groups	No. vaccinated^§^/Total (%)		Adjusted VE, % (95% CI)
	MIS-C case patients	Control patients	
**All controls**	5/102 (4.9)	65/181 (35.9)	91 (78–97)
Test-negative	5/102 (4.9)	34/90 (37.8)	92 (77–97)
Syndrome-negative	5/102 (4.9)	31/91 (34.1)	89 (70–96)
**Sensitivity analysis**			
MIS-C case patients with serologic evidence present^¶^	5/88 (5.7)	61/161 (37.9)	90 (75–96)

**Abbreviations:** MIS-C = multisystem inflammatory syndrome in children; VE = vaccine effectiveness.

* VE estimates were based on odds of antecedent vaccination in MIS-C case-patients versus controls adjusted for U.S. Census region, continuous age in years, sex, and race/ethnicity (non-Hispanic White, non-Hispanic Black, non-Hispanic multiple race/other, Hispanic of any race, or unknown). Firth penalized regression was used for models with six or fewer vaccinated cases.

^†^ Patients included vaccinated and unvaccinated persons aged 12–18 years enrolled from 24 pediatric hospitals in 20 states. *Northeast:* Boston Children’s Hospital (Massachusetts), Children's Hospital of Philadelphia (Pennsylvania), and Saint Barnabas Medical Center (New Jersey); *Midwest:* Akron Children’s Hospital (Ohio), Children’s Hospital and Medical Center: Nebraska (Nebraska), Children's Hospital of Michigan (Michigan), Children’s Mercy Kansas City (Missouri), Cincinnati Children’s Hospital Medical Center (Ohio), Lurie Children's Hospital of Chicago (Illinois), Mayo Clinic (Minnesota), Nationwide Children's Hospital (Ohio), and Riley Children's Hospital (Indiana); *South:* Arkansas Children’s Hospital (Arkansas), Children’s of Alabama (Alabama), Children's Healthcare of Atlanta (Georgia), Children’s Hospital of New Orleans (Louisiana), Medical University of South Carolina Children’s Health (South Carolina), Monroe Carell Jr. Children’s Hospital at Vanderbilt (Tennessee), Texas Children’s Hospital (Texas), University of Mississippi Medical Center (Mississippi), University of North Carolina at Chapel Hill Children’s Hospital (North Carolina), and University of Texas Southwestern Medical Center (Texas); *West:* Children’s Hospital Colorado (Colorado), Children’s Hospital Los Angeles (California), University of California San Diego-Rady Children’s Hospital (California), and University of California San Francisco Benioff Children’s Hospital Oakland (California).

^§^ COVID-19 vaccination status included the following two categories: 1) unvaccinated, defined as no receipt of any SARS-CoV-2 vaccine before hospitalization for current illness and 2) fully vaccinated, defined as receipt of both doses of a 2-dose Pfizer-BioNTech vaccination ≥28 days before illness onset.

^¶^ Analysis excluded 14 MIS-C case-patients who were positive by reverse transcription-polymerase chain reaction only with no serologic evidence of previous infection and 20 controls matched to these patients, given potential misclassification of patients with severe acute COVID-19.

## Discussion

During July–December 2021, a period of Delta variant predominance, a real-world evaluation of VE in 24 U.S. pediatric hospitals found that receipt of 2 doses of the Pfizer-BioNTech vaccine was associated with a high level of protection against MIS-C among patients aged 12–18 years who received their second vaccine dose ≥28 days before hospitalization. Most (95%) patients aged 12–18 years hospitalized with MIS-C were unvaccinated. No fully vaccinated patients with MIS-C required respiratory or cardiovascular life support, as opposed to 39% of unvaccinated MIS-C patients who did. A recent Overcoming COVID-19 hospital network investigation reported high VE (93% [95% CI = 83%–97%]) against COVID-19–related hospitalizations in persons aged 12–18 years (*6*). The current findings contribute to the growing body of evidence that vaccination is likely effective in preventing severe COVID-19–related complications in children, including MIS-C.

The findings in this report are subject to at least seven limitations. First, VE was not assessed against MIS-C attributed to specific variants; however, >99% of COVID-19 cases reported during July–December 2021 resulted from infections with the Delta variant (*9*). Second, VE against MIS-C attributed to the B.1.1.529 (Omicron) variant could not be assessed, given the timing of hospital admission of included patients relative to emergence of this variant in the United States. Third, timing of initial SARS-CoV-2 infection relative to vaccination could not be inferred, and this investigation cannot differentiate between protection from acquisition of SARS-CoV-2 infection versus protection against development of MIS-C after infection. Fourth, the timing at which protection against MIS-C is conferred after 2 doses of vaccine is unknown; some protection might be possible within 28 days of vaccination, and this investigation had insufficient power to evaluate VE for 1 dose of vaccine. Fifth, this analysis examines VE against MIS-C conferred only by the Pfizer-BioNTech vaccine. Sixth, although the hospital sites participating in this investigation covered a broad geographic area, the results of this analysis are not generalizable to the entire U.S. pediatric population. Finally, given the short time frame of enrollment, this analysis was not designed to evaluate waning immunity or duration of protection against MIS-C.

As of December 13, 2021, 52.3% of eligible U.S. children and adolescents aged 12–17 years had received the primary Pfizer-BioNTech 2-dose series (*10*). In a multistate hospital network, this real-world investigation found that receipt of 2 doses of Pfizer-BioNTech vaccine was strongly associated with prevention of MIS-C among adolescents. Children aged 5–11 years, who are now authorized to receive the Pfizer-BioNTech vaccine, represent the age group at highest risk for MIS-C (*1*,*3*). This analysis lends supportive evidence that vaccination of children and adolescents is highly protective against MIS-C and COVID-19 and underscores the importance of vaccination of all eligible children.

**Summary**

**What is already known about this topic?**

The Pfizer-BioNTech vaccine, currently authorized for persons aged ≥5 years, provides a high level of protection against severe COVID-19 in persons aged 12–18 years. Vaccine effectiveness against multisystem inflammatory syndrome in children (MIS-C), which can occur 2–6 weeks after SARS-CoV-2 infection, has remained uncharacterized.

**What is added by this report?**

Estimated effectiveness of 2 doses of Pfizer-BioNTech vaccine against MIS-C was 91% (95% CI = 78%–97%). Among critically ill MIS-C case-patients requiring life support, all were unvaccinated.

**What are the implications for public health practice?**

Receipt of 2 doses of Pfizer-BioNTech vaccine is highly effective in preventing MIS-C in persons aged 12–18 years. These findings further reinforce the COVID-19 vaccination recommendation for eligible children.

## Footnotes

* These authors contributed equally to this report.

† These senior authors contributed equally to this report.

§
https://www.fda.gov/emergency-preparedness-and-response/coronavirus-disease-2019-covid-19/comirnaty-and-pfizer-biontech-covid-19-vaccine

¶ In this context, the test-negative case-control design was used to compare the odds of previous Pfizer-BioNTech vaccine receipt among inpatients without evidence of SARS-CoV-2 infection with case-patients hospitalized for MIS-C. These control patients included those with respiratory virus infection who received a negative test result for SARS-CoV-2 infection (test-negative) or patients without symptoms compatible with COVID-19 (syndrome-negative), including fever, cough, shortness of breath, loss of taste, loss of smell, gastrointestinal symptoms, use of respiratory support for the acute illness, or new pulmonary findings on chest imaging consistent with pneumonia.

** This investigation included patients enrolled from 24 pediatric hospitals in 20 states: Alabama, Arkansas, California, Colorado, Georgia, Illinois, Indiana, Louisiana, Massachusetts, Michigan, Mississippi, Missouri, Nebraska, New Jersey, North Carolina, Ohio, Pennsylvania, South Carolina, Tennessee, and Texas.

††
https://overcomecovid.org/

§§ CDC recommendation for pediatric COVID-19 vaccine for children aged 5–11 years: https://www.cdc.gov/media/releases/2021/s1102-PediatricCOVID-19Vaccine.html

¶¶ The lower age bound for the study population was set at 12 years and 49 days to allow for the first vaccine dose on the patient’s 12th birthday, a second dose 21 days thereafter, and a 28-day window between the patient’s second dose and hospitalization for MIS-C.

*** CDC case definition criteria for MIS-C are available at https://www.cdc.gov/mis/mis-c/hcp/index.html. For the purposes of this analysis, all MIS-C case-patients were required to have laboratory evidence of current or recent infection (RT-PCR, antigen-, or antibody-based testing).

††† Vaccine effectiveness studies in the context of respiratory viruses most commonly include test-negative controls. Because of potential biases related to the selection of controls, including the potential for misclassification of test-negative patients due to false-negative tests, syndrome-negative controls were also included as a separate control group. Among the 91 syndrome-negative controls, 18 (20%) had no record of SARS-CoV-2 testing. The remaining syndrome-negative controls had a record of SARS-CoV-2 testing by RT-PCR or antigen and received negative test results.

§§§ Among the 102 MIS-C case-patients and 181 controls enrolled, 50 (49%) and 113 (62%), respectively, had information obtained through a combination of parent interview and medical records abstraction, while 52 (51%) case-patients and 68 (38%) control patients had information obtained solely through medical records abstraction.

¶¶¶ 45 C.F.R. part 46.102(l)(2), 21 C.F.R. part 56; 42 U.S.C. Sect. 241(d); 5 U.S.C. Sect. 552a; 44 U.S.C. Sect. 3501 et seq.

**** VE against MIS-C was also assessed comparing the odds of antecedent vaccination with the second dose of the Pfizer-BioNTech vaccine ≥14 days before hospital admission. Point estimates did not significantly differ from the primary analysis presented in this report. (VE after 14 days: 86%; 95% CI = 70%–93%.)
